# Distribution of Lewy-related pathology in the brain, spinal cord, and periphery: the population-based Vantaa 85 + study

**DOI:** 10.1186/s40478-022-01487-5

**Published:** 2022-12-12

**Authors:** Anna Raunio, Ville Kivistö, Mia Kero, Jarno Tuimala, Sara Savola, Minna Oinas, Eloise Kok, Kia Colangelo, Anders Paetau, Tuomo Polvikoski, Pentti J. Tienari, Henri Puttonen, Liisa Myllykangas

**Affiliations:** 1 Department of Pathology, University of Helsinki, Haartmaninkatu 3, PO Box 21, 00014 Helsinki, Finland; 2 HUS Diagnostic Center at Helsinki University Hospital, Helsinki, Finland; 3 Division of Clinical Neuroscience and Rehabilitation, Department of Neurosurgery, Ophthalmology and Otorhinolaryngology, University Hospital of North-Norway, Tromso, Norway; 4 Newcastle University Translational and Clinical Research Institute, University of Newcastle, Newcastle Upon Tyne, UK; 5 Translational Immunology, Research Programs Unit, University of Helsinki, Helsinki, Finland; 6 Department of Neurology, Helsinki University Hospital, Helsinki, Finland

**Keywords:** Neuropathology, Aged, 80 and over, Lewy body disease, Alpha-Synuclein, Spinal cord, Dorsal root ganglion, Adrenal gland

## Abstract

Evolving evidence has supported the existence of two anatomically distinct Lewy-related pathology (LRP) types. Investigation of spinal cord and peripheral LRP can elucidate mechanisms of Lewy body disorders and origins of synuclein accumulation. Still, very few unselected studies have focused on LRP in these regions. Here we analysed LRP in spinal cord, dorsal root ganglion, and adrenal gland in the population-based Vantaa 85 + study, including every ≥ 85 years old citizen living in the city of Vantaa in 1991 (n = 601). Samples from spinal cord (C6-7, TH3-4, L3-4, S1-2) were available from 303, lumbar dorsal root ganglion from 219, and adrenal gland from 164 subjects. Semiquantitative scores of LRP were determined from immunohistochemically stained sections (anti-alpha-synuclein antibody 5G4). LRP in the ventral and dorsal horns of spinal cord, thoracic intermediolateral column, dorsal root ganglion and adrenal gland were compared with brain LRP, previously determined according to DLB Consortium criteria and by caudo-rostral versus amygdala-based LRP classification. Spinal LRP was found in 28% of the total population and in 61% of those who had LRP in the brain. Spinal cord LRP was found only in those subjects with LRP in the brain, and the quantity of spinal cord LRP was associated with the severity of brain LRP (*p* < 0.001). Unsupervised K-means analysis identified two cluster types of spinal and brain LRP corresponding to caudo-rostral and amygdala-based LRP types. The caudo-rostral LRP type exhibited more frequent and severe pathology in spinal cord, dorsal root ganglion and adrenal gland than the amygdala-based LRP type. Analysis of specific spinal cord regions showed that thoracic intermediolateral column and sacral dorsal horn were the most frequently affected regions in both LRP types. This population-based study on brain, spinal and peripheral LRP provides support to the concept of at least two distinct LRP types.

**Supplementary Information:**

The online version contains supplementary material available at 10.1186/s40478-022-01487-5.

## Introduction

Alpha-synuclein (αSyn) rich Lewy bodies (LB) and Lewy neurites (LN) constitute Lewy-related pathology (LRP), the hallmark of Lewy body diseases, including dementia with Lewy bodies (DLB) and Parkinson’s disease with (PDD) and without dementia (PD) [36, 49]. In addition to the brain, LRP has been found in spinal cord [2, 3, 12, 18, 26, 38] and in various regions of the peripheral nervous system (PNS), such as sensory neurons of the dorsal root ganglia (DRG) [38, 48] and the adrenal gland [11, 12].

Evolving evidence from imaging and animal studies have supported a prion-like propagation hypothesis of LRP progression [13, 20, 21, 33, 37, 43, 45], with human and animal studies suggesting that LRP exhibits two anatomically distinct progression patterns [4, 13, 15, 31, 42, 46]. 1) In the caudo-rostral LRP type, LRP has been suggested to originate in the enteric nervous system and to proceed rostrally to brainstem and other parts of the brain [5, 8, 9, 41]. 2) In the amygdala-based LRP type, LRP has been suggested to occur first in amygdala/olfactory regions of the brain, from which LRP then progresses to neocortical and brainstem regions [4, 31, 42, 44]. However, it is still unclear how spinal cord and PNS regions are involved in the two progression models of these diseases.

Few studies have analysed LRP in spinal cord regions [2, 6, 9, 26, 38], most of them focused on the total burden of LRP at various spinal cord levels. A hospital-based Japanese study also analysed the anatomic distribution of LRP at various levels of the spinal cord: in the thoracic sympathetic intermediolateral column (IML), intermediate zones of cervical, lumbar, and sacral levels, as well as dorsal and ventral horns at all four levels [38]. Although two population- or community-based studies [6, 26] have reported the total burden of αSyn at different spinal cord levels, to our knowledge no study has focused on the anatomical distribution of spinal cord and PNS LRP in an unselected study setting, nor in the context of caudo-rostral versus amygdala-based LRP types.

To address these issues in the population-based Vantaa 85 + study, herein we have semiquantitatively studied the occurrence of LRP in the ventral and dorsal horns at four levels of spinal cord, in sympathetic intermediolateral column at the thoracic level, adrenal gland and lumbar DRG. We show here for the first time that the distribution of spinal and peripheral LRP is more frequent and severe in the caudo-rostral compared to the amygdala-based LRP type.

## Materials and methods

### Study subjects

The Vantaa 85 + study cohort includes every at least 85-year-old citizen, who lived in the city of Vantaa, Finland on the first of April 1991 (n = 601). General and neuropathological autopsy was performed whenever possible, and eventually 304 (51%) underwent consented general and neuropathological postmortem examination (the extent of postmortem examination was not influenced by the cause of death). For this study, brains from 304, spinal cord from 303, samples of the lumbar DRG from 219 and samples of the adrenal gland from 164 study participants were available. The variation in sample numbers was mainly due to technical problems. Demographics of the study subjects have been published before, as well as details of previously determined clinical, genetic, and neuropathological variables [19, 25, 27, 29–32, 34]. There were no significant differences in age at death or sex in the neuropathologically examined subpopulation compared with the whole study population [27].

### Procedures

LRP was detected as pathologically misfolded αSyn by immunohistochemical staining using mouse monoclonal anti-αSyn antibody (clone 5G4, 1:3000, AJ Roboscreen or Millipore) on 5 µm formalin-fixed paraffin-embedded (FFPE) sections of the spinal cord at cervical (C6-7), thoracic (TH3-4), lumbar (L3-4) and sacral (S1-2) levels, as well as DRG at the lumbar level and adrenal gland. Because the formalin-fixation time varied, including prolonged fixation periods, the 5G4 antibody was chosen and found to successfully stain sections using a suggested citric acid epitope retrieval and formic acid pretreatment [22]. Immunostaining was performed with the EnVision Flex Visualisation System (Agilent Dako). First, heat induced epitope retrieval (HIER) treatment was performed in a TintoRetriever pressure cooker using Dako Low pH solution. After buffer solution washes, sections were then pretreated for 5 min in 100% formic acid and the staining procedure performed using LabVision equipment following the EnVision Flex Visualisation protocol. Pathologically aggregated αSyn was screened by a pathologist blinded to other data utilising manual (by eye) judgment for each section using a low power microscopic field (100X magnification). 200X and 400X magnifications were used for verifying results. In each spinal cord section having LRP the area of dorsal horn (Rexed laminae I-VI) and ventral horn (Rexed laminae VIII-IX) were analysed (the side with more LRP was chosen for analysis). At the thoracic (TH3-4) level, the IML was additionally evaluated. LBs were counted in each investigated region within sections and those exceeding the size of erythrocytes were included in counted particles. LNs (dot-like immunopositive structures not included) were recorded either as present or not present. LRP was further divided into four semiquantitative stages 0 = none, 1 = mild (1 LB and/or LN), 2 = moderate (2–3 LB) and 3 = severe (over 4 LB) according to modified DLB Consortium scoring guidelines [24]. The association of spinal cord LRP was compared to previously categorised DLB Consortium LRP types (olfactory-only, amygdala-predominant, brainstem-predominant, limbic, diffuse neocortical, and non-classifiable) and previously suggested LRP progression patterns (caudo-rostral type and amygdala-based type, Additional file 1: Table S1) [19, 23, 31]. For DRG sections also granular αSyn positivity was included, in addition to LB and LN pathology, with αSyn positivity determined as either present or absent. For the adrenal gland, LRP was assessed as either present or absent in the adrenal medulla or in nervous structures in the surrounding adipose tissue. A dichotomised classification was chosen for αSyn pathology in the DRG or adrenal gland samples because these pathologies were generally relatively modest, and the number of samples collected from these regions was lower than in other regions.

### Statistical analysis

All statistical analyses were assessed using R 4.0.1 (https://www.r-project.org) and / or IBM SPSS Statistics version 27. The associations of continuous variables were assessed using the independent-Samples Mann–Whitney U Test. The association in nominal or categorical variables were assessed using Fisher’s exact test. Mantel–Haenszel test was used for assessing the trend of spinal cord dorsal, ventral or intermediolateral column, DRG and adrenal gland αSyn/LRP between DLB Consortium LRP types. An ordinal regression random effect model was used to assess the strength of a sacral to cervical gradient in the dorsal horn and ventral horn LRP. An unsupervised K-means cluster analysis was used to assess patterns of LRP as previously described with nine clusters judged optimal using the “elbow method” [16, 31], however for these analyses semiquantitative classes 3 (severe) and 4 (very severe) were combined. *p*-values under 0.05 were considered statistically significant. *p*-values were adjusted for multiple comparisons using Bonferroni correction.

## Results

### All cases with spinal LRP have brain LRP

Altogether 85 individuals had LRP in at least one of the investigated spinal cord regions, which constitutes 28% of all 303 neuropathologically investigated individuals and 61% of the 139 cases with brain LRP. It is of note that all cases with spinal cord LRP showed LRP in at least one brain region (Table 1). Table 2 summarises the characteristics of study subjects stratified according to presence versus absence of spinal cord LRP with concomitant brain LRP. All *p*-values of comparisons between subgroups are shown in Additional file 1: Table S2. Subjects with concomitant brain and spinal cord LRP had significantly more neuron loss in the substantia nigra (Bonferroni corrected *p*-value = 9.0045E-6) when compared with subjects showing no LRP. The high Braak NFT stage (Bonferroni corrected *p*-value = 0.02574) showed a significant association when comparing those cases which showed only brain but no spinal cord LRP, versus cases with no LRP.

**Table 1:** Number of the neuropathologically investigated cases from the Vantaa 85 + study with LRP in spinal cord and/or brain

		LRP in spinal cord^a^	
		No	Yes
LRP in brain^b^	no	164	0
	yes	54	85

*p* = 2.3423E-38

LRP Lewy -related pathology

^a^LRP detected in any of the investigated spinal cord regions: dorsal horn and ventral horn at the cervical 6–7, thoracic 3–4, lumbar 3–4 and sacral 1–2 levels and intermediolateral column at thoracic 3–4 level, one case with no spinal cord regions available was excluded from the analysis

^b^LRP detected in any of the investigated brain regions: dorsal motor nucleus of Vagus (dmV) of medulla, locus coeruleus (LC) of pons, substantia nigra, amygdala, entorhinal cortex, cingulate gyrus, temporal cortex, frontal cortex, parietal cortex, anterior olfactory nucleus (AON) and peripheral olfactory bulb/peduncle (brain LRP data published previously [19, 31]). 15 cases found to have LRP only in olfactory bulb/peduncle were included [19]. Cases with missing olfactory regions but all other brain regions negative were considered negative[19]

**Table 2:** Characteristics of the investigated subgroups from the Vantaa 85 + study

	No LRP in any brain^a^ or spinal cord^b^ region (n = 164)	LRP in any brain^a^ region but no spinal cord^b^ LRP (n = 54)	Concomitant brain^a^ and spinal cord^b^ LRP (n = 85)
Women	138 (84%)	49 (91%)	64 (75%)
Age at death, yrs (SD)	92.3 (3.4)	92.6 (3.3)	92.4 (4.5)
Dementia	94 (57%)	39 (72%)	62 (73%)
Age at onset of dementia, yrs (SD)	87.3 (4.3)	86.5 (4.0)	87.5 (5.3)
Duration of dementia, yrs (SD)	5.1 (3.7)	6.2 (4.0)	5.0 (3.6)
*APOE4*	41 (27%)	24 (46%)	25 (33%)
SN neuronal loss (moderate-severe)	51 (31%)	27 (50%)	56 (66%)^***^
Braak NFT stage (V-VI)	45 (28%)	30 (56%)^*^	31 (37%)
Thal phases 4–5	106 (65%)	42 (78%)	62 (73%)
CERAD score (moderate-frequent)	100 (61%)	40 (74%)	60 (71%)

LRP Lewy-related pathology, SN substantia nigra, NFT neurofibrillary tangle

^a^LRP detected in any of the investigated brain regions: dorsal motor nucleus of Vagus (dmV) of medulla, locus coeruleus (LC) of pons, substantia nigra, amygdala, entorhinal cortex, cingulate gyrus, temporal cortex, frontal cortex, parietal cortex, anterior olfactory nucleus (AON) and peripheral olfactory bulb/peduncle (brain LRP data published previously [19, 31]). 15 cases found to have LRP only in olfactory bulb/peduncle [19] were included in the LRP in any brain region, no spinal LRP -group

^b^LRP detected in any of the investigated spinal cord regions: dorsal horn and ventral horn at cervical 6–7, thoracic 3–4, lumbar 3–4 and sacral 1–2 levels and the intermediolateral column at thoracic 3–4 level, one case with no spinal cord regions available was excluded from the analysis

The groups with either brain or concomitant brain and spinal cord LRP were individually compared with the no LRP group. Significant differences after Bonferroni correction are marked with asterisks, raw *p*-values are shown in Additional file 1: Table S2

Demographics [30] of the study subjects have been published previously as well as clinical [27], genetic [25] and neuropathological variables (SN neuronal loss [27], Braak NFT stage and Thal phases [34], CERAD score [30])

### The quantity of spinal cord LRP shows a gradient in dorsal horn

**Fig. 1:**
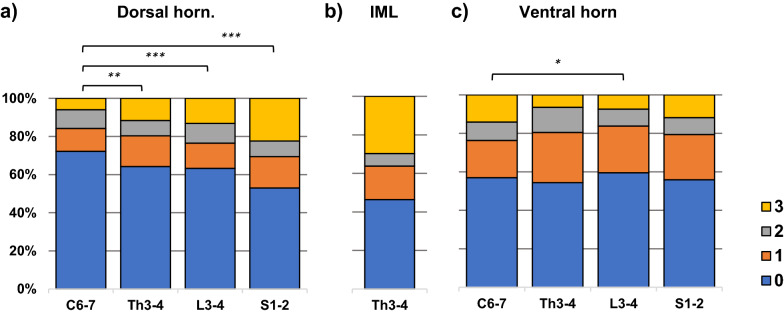
Distribution of semiquantitative LRP scores in subjects with brain LRP in at least one investigated brain region (n = 139). **a** Dorsal horn and **c** ventral horn spinal cord regions at four spinal cord levels (C6-7, TH3-4, L3-4, S1-2). **b** Thoracic IML region (TH3-4). Based on the ordinal regression random effect model, a sacral to cervical gradient in the strength of associations was seen in dorsal horn but not in ventral horn LRP (the numerical data shown in Additional file 1: Table S4). *p-value < 0.05, ** *p*-value < 0.01, ***p-value < 0.001 after Bonferroni correction

Figure 1 and Additional file 1: Table S3 show the semiquantitative spinal cord LRP scores in dorsal horn (C6-7, TH3-4, L3-4, S1-2), thoracic (TH3-4) IML, which contains preganglionic sympathetic neurons, and ventral horn (C6-7, TH3-4, L3-4, S1-2). The amount of LRP was highest in the thoracic IML region (only TH3-4 region was analysed). The dorsal horn exhibited a gradient where the sacral cord had the highest amount of LRP and cervical cord the lowest (Fig. 1a, Additional file 1: Table S4). This was seen particularly when the severe (3) semiquantitative category was examined. The ventral horn did not show a similar trend even though there was a significant difference when comparing the lumbar and cervical ventral horn LRP (Fig. 1c and Additional file 1: Table S4).

### Severity of spinal cord LRP associates with the DLB Consortium types

**Fig. 2:**
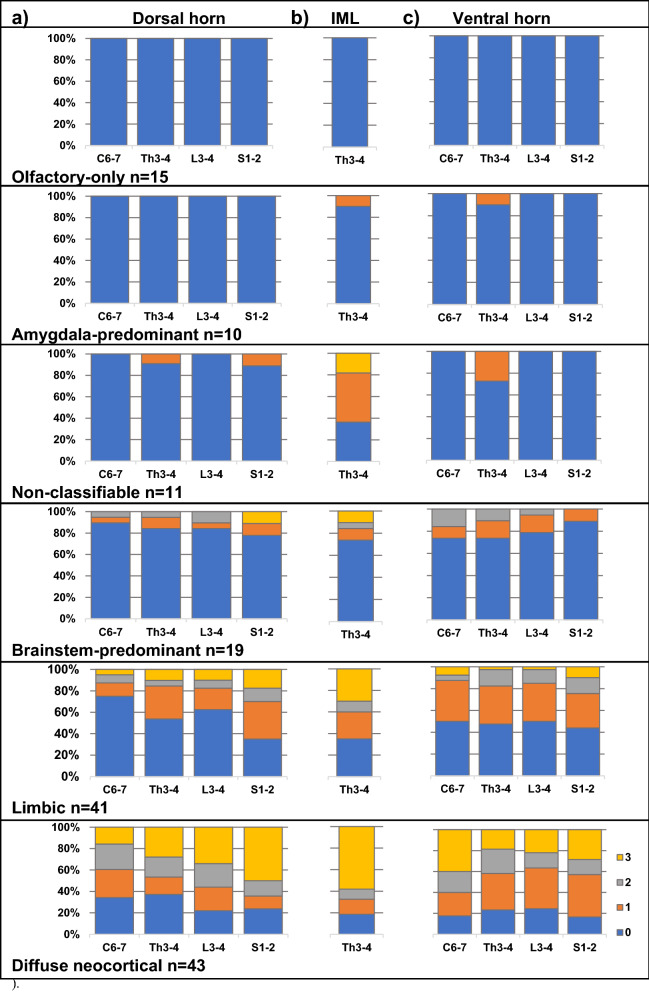
The frequency of semiquantitative spinal cord LRP scores stratified according to previously determined DLB Consortium LRP types n = 139 [19, 31]. The frequency of spinal cord LRP increased when more severe brain LRP was found (Linear-by-Linear Association Mantel–Haenszel Test, Additional file 1: Table S5)

Figure 2 shows the semiquantitative LRP scores in dorsal and ventral horns at different spinal cord levels and in the thoracic IML, stratified according to the DLB Consortium types. In general, the stronger the pathology in the brain, the more severe pathology was seen in the spinal cord (Linear-by-Linear Association Mantel–Haenszel Test, Additional file 1: Table S5). The diffuse neocortical LRP type showed the most severe spinal cord LRP whereas the non-classifiable, olfactory-only and amygdala-predominant LRP types showed no or only very mild spinal cord LRP. The thoracic IML showed the most frequent LRP in every DLB Consortium LRP type apart from the olfactory-only type, which exhibited no spinal LRP at all.

### K-means analysis reveals two distinct types of LRP clusters

**Fig. 3:**
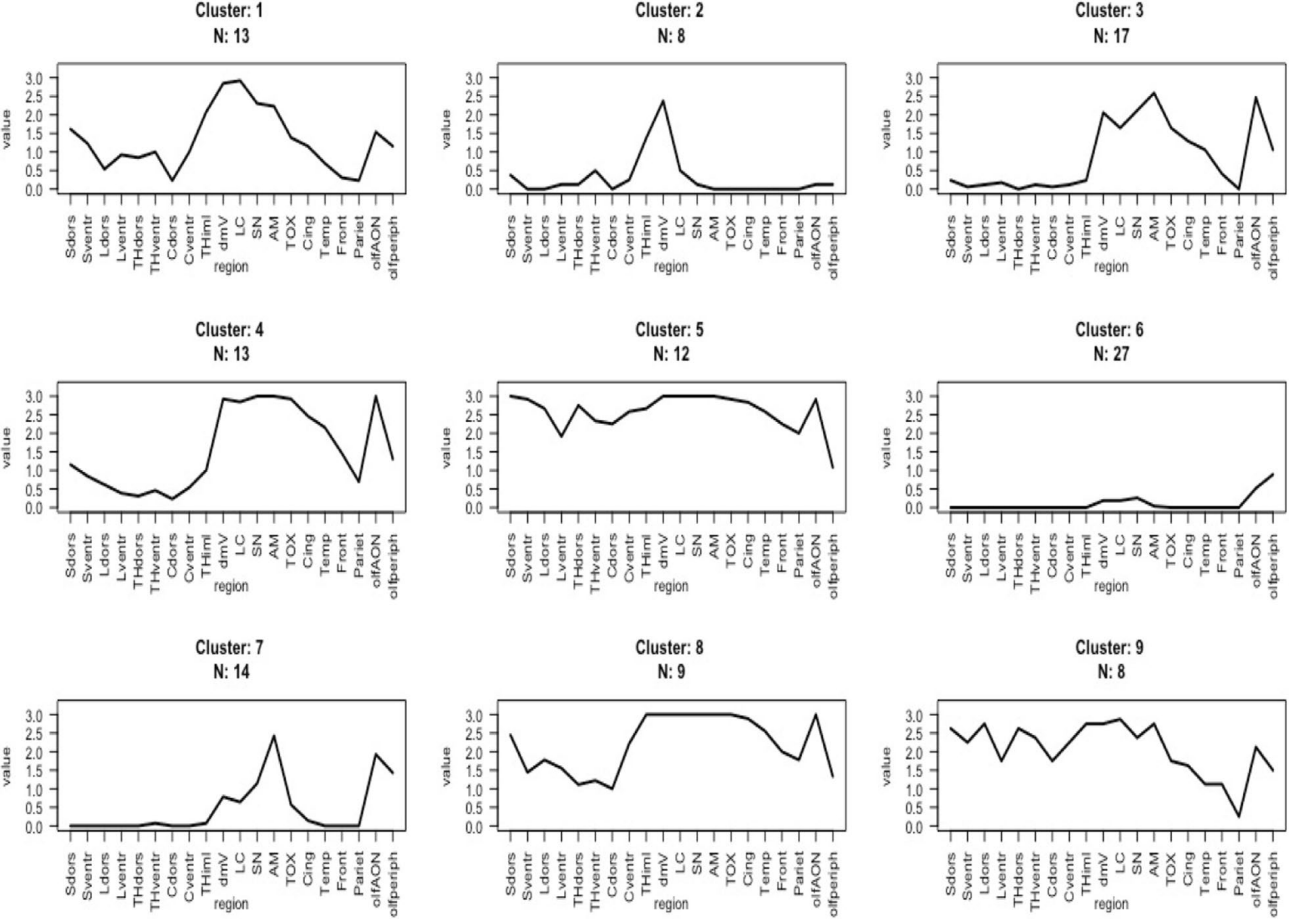
K-means cluster analysis reveals distinct LRP subtypes. Only cases with data available for each spinal cord and brain region were included n = 121 (18 cases were excluded because of missing data in any of the regions). K-means cluster analysis was performed as previously described [31], however for these analyses semiquantitative classes 3 (severe) and 4 (very severe) were combined

To analyse the potential presence of distinct LRP subtypes we then performed an unsupervised K-means cluster analysis (Fig. 3). This analysis revealed two main kinds of clusters of spinal and brain LRP. In one type (clusters 3,4, and 7) there was none or only modest spinal cord LRP and the highest peak of LRP was observed in the amygdala region. In the other type abundant or moderate spinal cord LRP was seen concomitantly with brain LRP, peaking in the brainstem regions (clusters 1, 2, 5, 8, and 9). In addition, one of the clusters (cluster 6) showed subjects with only modest LRP in olfactory and/or brainstem regions.

### Caudo-rostral LRP type shows more spinal cord LRP than amygdala-based LRP type

**Fig. 4:**
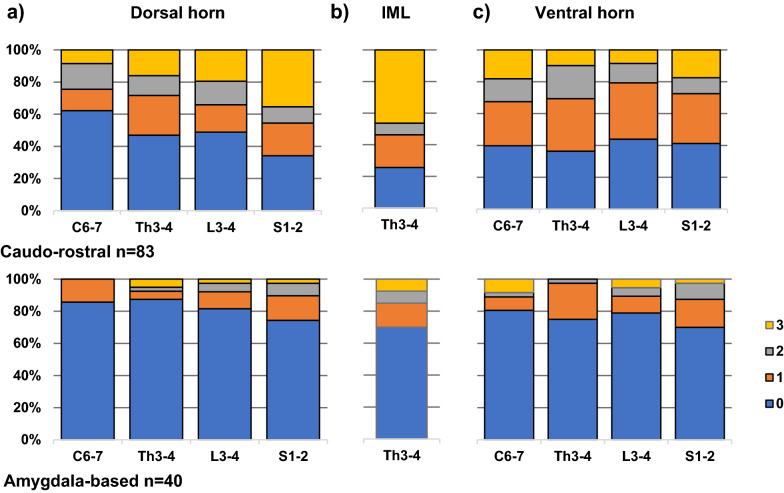
Distribution of semiquantitative spinal cord LRP in caudo-rostral versus amygdala-based LRP types. Includes all cases with spinal cord regions available that were categorised into caudo-rostral and amygdala-based LRP types in previously published work [31], with olfactory-only cases [19] n = 15, excluded from analysis. The frequency of spinal cord LRP was higher in subjects with the caudo-rostral LRP type, compared to those with the amygdala-based LRP type in all anatomical regions (Fisher’s exact test uncorrected *p* < 0.01, Bonferroni corrected p < 0.09) (Additional file 1: Table S6). The sacral dorsal horn and thoracic IML showed the most significant *p*-values after Bonferroni correction ((*p* = 0.000216 and *p* = 0.000027, respectively)

**Fig. 5:**
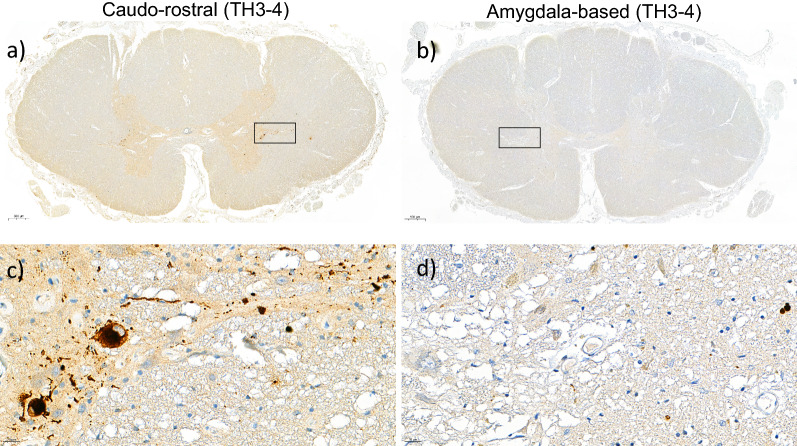
Two examples of thoracic spinal cord sections immunostained with anti-αSyn-antibody clone 5G4. Both cases were classified according to the DLB Consortium as diffuse neocortical LRP type. **a** In a case with caudo-rostral LRP type, LRP was severe in IML, dorsal and ventral horns (20×). **b** In a case with amygdala-based LRP type, only modest pathology was seen in the IML and ventral horn (20×). In **c** and **d** high-power field (400×) of IML marked by the rectangle in (**a**) and (**b**) is shown, respectively

We next analysed the distribution of spinal cord LRP in caudo-rostral (n = 83) and amygdala-based (n = 40) LRP types, which we previously described [31]. Although the same anatomical regions were affected in both LRP types, the frequency of spinal cord LRP was higher in subjects with the caudo-rostral LRP type, when compared to those with the amygdala-based LRP type in all anatomical regions (Fisher’s exact test uncorrected *p* < 0.01, Bonferroni corrected *p* < 0.09). The sacral dorsal horn and thoracic IML showed the most significant *p*-values (Bonferroni corrected *p* = 0.000216 and *p* = 0.000027, respectively). The frequency of thoracic IML LRP was two times higher in caudo-rostral versus the amygdala-based LRP type (67% vs. 33%) (Fig. 4, Additional file 1: Table S6). Figure 5 illustrates this difference by presenting thoracic sections immunostained with αSyn antibody (5G4) from a case with the caudo-rostral LRP type (Fig. 5a, c) and a case with the amygdala-based LRP type (Fig. 5b, d).

### Lumbar DRG αSyn positivity

**Fig. 6:**
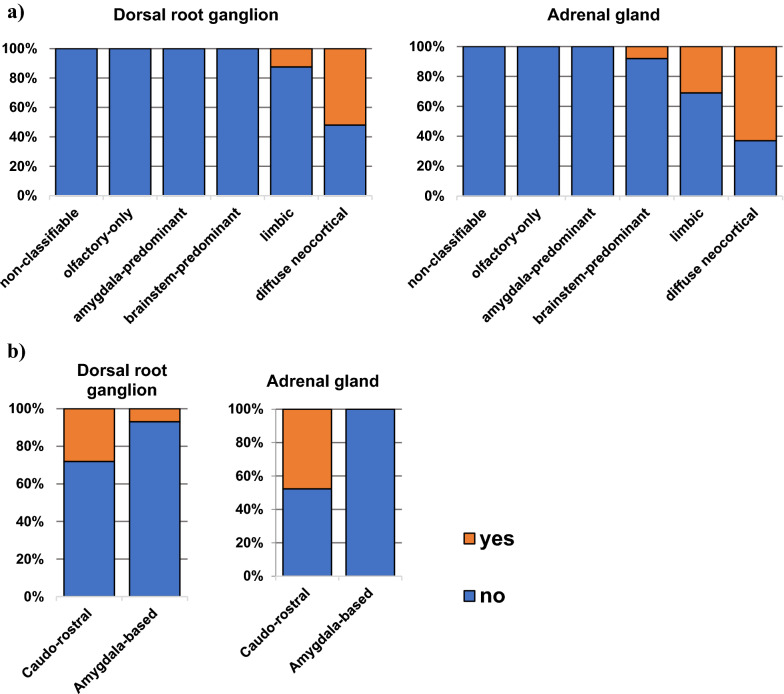
Lumbar DRG and adrenal gland αSyn positivity compared with **a** DLB Consortium LRP types and **b** LRP progression types. DRG and adrenal gland pathology was associated with DLB Consortium types. αSyn pathology was more frequent in caudo-rostral versus amygdala-based LRP type (numerical data shown in Additional file 1: Table S8)

In lumbar DRG αSyn positivity was detected in 9% of all investigated samples (19/219). In all cases with lumbar DRG αSyn positivity, LRP was present in the brain and spinal cord. Lumbar DRG αSyn positivity was only seen in those who had DLB Consortium LRP type limbic (4 subjects) or diffuse neocortical LRP type (15 subjects). 28% (16/57) of subjects with the caudo-rostral LRP type and 7% (2/29) of those with the amygdala-based LRP type had αSyn positive lumbar DRG samples (Fig. 6 and Additional file 1: Table S7).

### Adrenal gland LRP

Adrenal gland LRP was detected in 22/164 (13%) of subjects with adrenal gland samples available. All cases with LRP in the adrenal gland also had LRP in the brain and spinal cord. 21 subjects of these were previously categorised as the caudo-rostral LRP type and one adrenal gland positive subject belonged to the all-highest category [31]. According to DLB Consortium classification, one was brainstem-predominant, four limbic and the remaining 17 of the diffuse neocortical LRP type (Fig. 6 and Additional file 1: Table S7).

## Discussion

Lewy body diseases were long considered to affect only brain, but in recent decades it has been increasingly acknowledged that these diseases affect the whole nervous system including PNS, possibly explaining some non-motor symptoms (NMS), such as constipation, falls, dysregulation of blood pressure and pain, known to be associated with these diseases [14]. Analysis of spinal cord and peripheral LRP can elucidate the pathophysiological basis of these NMS and may give important clues for progression patterns of LRP and the origins of LRP accumulation. However, there are few histological sample collections and even fewer unselected samples where the spinal cord and peripheral LRP can be investigated. Here we have investigated the quantity of LRP in these regions in an unselected elderly population (Vantaa 85 + study) and report the following main results. 1) Spinal pathology is found only in those subjects with LRP in the brain. 2) The quantity of spinal cord LRP associates with the severity of brain LRP. 3) The caudo-rostral LRP type is more strongly associated with spinal cord, DRG and adrenal gland pathology than the amygdala-based LRP type, further supporting the view of at least two distinct progression patterns of LRP. 4) LRP in the spinal cord accumulates in the same anatomical areas of the spinal cord in both LRP progression types.

Although it is difficult to compare the results of different studies due to variation in study design, inclusion criteria and differences in methodology and sampling, our results confirm several previous findings. We found no cases with LRP restricted to spinal cord, in line with several previous studies [6, 9, 38]. In addition, the severity of brain LRP was associated with more severe spinal cord LRP, also consistent with previous studies [26]. Furthermore, we report that 28% of the whole population had spinal cord LRP and 61% of those that had brain LRP had concomitant spinal cord LRP. In several community-based and hospital-based studies the frequency has been reported to be about 20% [3, 6, 18, 38] and 50–65% of subjects with brain LRP have concomitant spinal cord LRP, and thus our findings can be interpreted to be in accordance with these results. [2, 38]. In accordance with our previous study [26], subjects with concomitant brain and spinal LRP were more frequently found to have substantia nigra neuron loss when compared with subjects with no LRP, possibly reflecting more severe LRP in subjects with concomitant brain and spinal LRP. In line with Sumikura et al., we found the sacral-cervical gradient of LRP, most pronounced in the dorsal horn [38]. Previous studies have shown that thoracic IML is the spinal region where LRP occurs in PD the earliest [9, 40]. Consistent with this we found that the thoracic IML was the anatomical spinal cord region with the most severe LRP.

The main novel finding of this study is that the presence and severity of spinal cord LRP is strongly associated with the caudo-rostral versus amygdala-based LRP types, highlighted by the results of the objective K-means cluster analysis. Although the same anatomical regions of spinal cord were affected in both LRP types, the caudo-rostral type more frequently showed spinal cord LRP even in cases with relatively mild brain LRP, whereas the amygdala-based LRP type mainly showed spinal LRP associated with severe brain LRP. Thus, it appears that with the caudo-rostral type, spinal cord LRP accumulation occurs earlier and, in the amygdala-based type it may represent a late-stage phenomenon. The fact that none of our 15 cases [19] with DLB Consortium olfactory-only LRP type showed spinal cord LRP is consistent with the view of two distinct origins of LRP. To the best of our knowledge, the spinal cord LRP in olfactory-only cases has previously been investigated in only two studies, where altogether three cases with olfactory-only LRP exhibited no spinal cord LRP, in line with our findings [2, 9]. Thus, our unselected data on 15 olfactory-only cases are an important extension to the previous literature.

In addition to CNS pathology, we investigated DRG and adrenal gland αSyn positivity and found that 9% and 13% of subjects had positivity in these areas, respectively. These results are in accordance with previous studies [11, 38, 48]. We report here that both pathologies are mainly found in cases with the caudo-rostral LRP type. In the Japanese Brain Bank study all cases with adrenal gland LRP also had concomitant brain LRP [41], similar to our findings. However, the Japanese group has reported the presence of 9 individuals with LRP present in PNS only (either in pericardial adipose tissue or thoracic sympathetic ganglion) [41] suggesting that LRP can originate outside the CNS. Even though this phenomenon has been reported in animal models [17, 45], further studies on larger representative human materials are needed to investigate its frequency in elderly populations.

Although it is not possible to directly investigate the mechanisms of progression of αSyn pathology in a postmortem study, indirect evidence for these mechanisms can be suggested. First, in our present study the most severe LRP was found in thoracic IML and dorsal sacral regions. In rodents, expression levels of αSyn have been found to be particularly pronounced in the spinal cord laminas I, II, VII and X located in the dorsal horn and intermediate zone regions [1, 47]. Previous studies on mice and humans have indicated that regional expression levels of physiological αSyn and vulnerability to LRP may be associated [10, 39], consistent with the prion theory of αSyn progression [20, 21, 37, 43]. In humans, expression levels of αSyn in different anatomical regions and levels of spinal cord have not been investigated to our knowledge. It remains to be further studied if different expression profiles are associated with the anatomical distribution of LRP and the sacral-cervical gradient in the dorsal horns, as reported by us and others [38]. Second, it has been pointed out that in Lewy body diseases, in contrast to other neurodegenerative diseases, the peripheral autonomic nervous system is significantly involved [28]. Our results are in line with this notion. In particular, the afferent visceral system may be involved in the progression of spinal cord LRP, as dorsal horn and IML are central anatomic regions in this system. Third, our data indicates that there may be temporal differences in the accumulation of spinal cord LRP in distinct LRP progression types. Imaging studies have also suggested two distinct temporal models of αSyn pathology progression [13]. Recent reports have indicated that there may be more structural heterogeneity of the αSyn protein in different synucleinopathies than previously understood [7, 35, 50] and it remains to be investigated whether this heterogeneity is linked to distinct temporal patterns of αSyn pathology progression.

Our study is one of the very few neuropathologically examined population-based studies of the very elderly [51], but it has limitations. It is focused on a very elderly unselected Finnish population mostly representing women with a mean age of death over 92 years, which can be considered ‘survivors’. Although the study is clinico-pathological, limited clinical data is available e.g. due to multiple diseases of very elderly subjects, and the main data of this study is focused on the cross-sectional collection of neuropathological samples at death. However, the wide non-hierarchical sampling protocol has enabled us to thoroughly study the distribution of LRP in the CNS and we have previously reported LRP to be frequent in this population [31]. It is noteworthy that spinal cord samples at four levels in an unselected study design of very elderly are rarely available for investigation in a population-based setting. The systematic sampling protocol was most uniform in the brain and spinal cord regions, but sampling of the peripheral regions was not similarly standardised (for example adrenal gland samples were mostly collected from one side only and from only about half of study subjects). Because of this it is possible we have missed some cases with peripheral LRP and no LRP in the CNS.

## Conclusions

Our population-based data supports the existence of at least two different LRP types. The caudo-rostral cases showed more frequent and severe spinal and peripheral LRP, whereas in the amygdala-based cases these pathologies were milder and appeared to occur at later stages of disease.

## Supplementary Information

**Additional file 1**.**Supplementary table 1**. Demographic characteristics of LRP types. **Supplementary table 2.** Results of the statistical analyses of Table 2. **Supplementary table 3.** The characteristics of semiquantitative scores of spinal cord LRP in different anatomical regions. **Supplementary table 4.** Results of the statistical analyses of Figure 1. **Supplementary table 5**. The results of the statistical analyses of Figure 2. **Supplementary table 6**. The results of the statistical analyses of Figure 4.  **Supplementary table 7.** Frequency of adrenal gland and lumbar dorsal root ganglion LRP compared with DLB Consortium LRP types and LRP progression patterns. **Supplementary table 8.** The results of statistical analyses of Figure 6.

## Data Availability

The datasets used and/or analysed during the current study are available from the corresponding author upon reasonable request.

## Abbreviations

αSyn: Alpha-synuclein
AD: Alzheimer’s dementia
ALS: Amyotrophic lateral sclerosis
AON: Anterior olfactory nucleus
*APOE4*: Apolipoprotein E4
CERAD: The consortium to establish a registry for alzheimer's disease
CNS: Central nervous system
DLB: Dementia with Lewy bodies
dmV: Dorsal motor nucleus of Vagus
DRG: Dorsal root ganglion
e.g.: Exempli gratia, for example
FTD: Frontotemporal dementia
HIER: Heat induced epitope retrieval
IML: Intermediolateral column
LB: Lewy body
LC: Locus coeruleus
LN: Lewy neurite
LRP: Lewy-related pathology
NFT: Neurofibrillary tangle
NMS: Non-motor symptoms
PD: Parkinson’s disease
PDD: Parkinson’s disease with dementia
PNS: Peripheral nervous system
SD: Standard deviation
SN: Substantia nigra
yrs: Years
