# Supplementary material for: Distribution of Lewy-related pathology in the brain, spinal cord, and periphery: the population-based Vantaa 85 + study
Source: Acta Neuropathol Commun. 2022 Dec 12;10:178. doi: 10.1186/s40478-022-01487-5 (PMC9743559; doi:10.1186/s40478-022-01487-5)
Supplement: Supplementary file 1 — Additional file 1.Supplementary table 1. Demographic characteristics of LRP types. Supplementary table 2. Results of the statistical analyses of Table 2. Supplementary table 3. The characteristics of semiquantitative scores of spinal cord LRP in different anatomical regions. Supplementary table 4. Results of the statistical analyses of Figure 1. Supplementary table 5. The results of the statistical analyses of Figure 2. Supplementary table 6. The results of the statistical analyses of Figure 4. Supplementary table 7. Frequency of adrenal gland and lumbar dorsal root ganglion LRP compared with DLB Consortium LRP types and LRP progression patterns. Supplementary table 8. The results of statistical analyses of Figure 6. [file 40478_2022_1487_MOESM1_ESM.pdf]

Supplementary material

Distribution of Lewy-related pathology in the brain, spinal cord, and periphery: the population-based Vantaa 85+ study

Anna Raunio, Ville Kivistö, Mia Kero, Jarno Tuimala, Sara Savola, Minna Oinas, Eloise Kok, Kia Colangelo, Anders Paetau, Tuomo Polvikoski, Pentti J. Tienari, Henri Puttonen* and Liisa Myllykangas*

Anna Raunio: Department of Pathology, University of Helsinki and HUS Diagnostic Center at Helsinki University Hospital, Helsinki, Finland, anna.raunio@helsinki.fi

Ville Kivistö: Department of Pathology, University of Helsinki, ville.kivisto@helsinki.fi

Mia Kero: Department of Pathology, University of Helsinki and HUS Diagnostic Center at Helsinki University Hospital, Helsinki, Finland, mia.kero@hus.fi

Jarno Tuimala: Department of Pathology, University of Helsinki, jtuimala@gmail.com

Sara Savola: Department of Pathology, University of Helsinki and HUS Diagnostic Center at Helsinki University Hospital, Helsinki, Finland, sara.savola@helsinki.fi

Minna Oinas: Department of Pathology, University of Helsinki Helsinki, Finland, and Division of Clinical Neuroscience and Rehabilitation, Department of Neurosurgery, Ophthalmology and Otorhinolaryngology, University Hospital of North-Norway, Tromso, Norway, minna.oinas@helsinki.fi

Eloise Kok: Department of Pathology, University of Helsinki and HUS Diagnostic Center at Helsinki University Hospital, Helsinki, Finland, eloise.mikkonen@helsinki.fi

Kia Colangelo: Department of Pathology, University of Helsinki, Helsinki, Finland, kia.colangelo@helsinki.fi

Anders Paetau: Department of Pathology, University of Helsinki and HUS Diagnostic Center at Helsinki University Hospital, Helsinki, Finland, anders.paetau@helsinki.fi

Tuomo Polvikoski: Newcastle University Translational and Clinical Research Institute, University of Newcastle, Newcastle upon Tyne, United Kingdom, t.m.polvikoski@newcastle.ac.uk

Pentti J. Tienari: Translational Immunology, Research Programs Unit, University of Helsinki, and

Department of Neurology, University of Helsinki and Helsinki University Hospital, Helsinki, Finland, pentti.tienari@hus.fi

Henri Puttonen: Department of Pathology, University of Helsinki and HUS Diagnostic Center at Helsinki University Hospital, Helsinki, Finland, henri.puttonen@helsinki.fi

Liisa Myllykangas: Department of Pathology, University of Helsinki and HUS Diagnostic Center at Helsinki University Hospital, Helsinki, Finland, liisa.myllykangas@helsinki.fi

* Equal contribution

Corresponding author

Liisa Myllykangas, MD, PhD, Associate professor of neuropathology, Consultant neuropathologist, PO Box 21 (Haartmaninkatu 3), 00014 University of Helsinki
Email: liisa.myllykangas@helsinki.fi

Supplementary tables

Supplementary table 1. Demographic characteristics of LRP types [4].

Variable	Caudo-rostral n=83	Amygdala-based n=40	*p*-values
Women	63 (76%)	35 (88%)	0.157566
Age at death, yrs (SD)	92.6 (4.2)	92.3 (4.0)	0.816417
Dementia	52 (63%)	37 (93%)	**0.000448
Age at onset of dementia, yrs (SD)	88.5 (4.5)	85.3 (5.2)	0.016019
Duration of dementia, yrs (SD)	4.2 (3.4)	6.9 (3.6)	**0.000215
APOE4	24 (31%)	22 (63%)	*0.001843
SN neuronal loss (moderate-severe)	47 (57%)	29 (73%)	0.042488
Braak NFT stage (V-VI)	18 (22%)	33 (83%)	***2.7203E-10
Thal phases 4-5	53 (64%)	39 (98%)	***0.000040
CERAD score (moderate-frequent)	50 (60%)	39 (98%)	***0.000019

Our guidelines for categorising LRP progression pattern according to the previously published systematic anatomical scoring [4] were in brief
(A) Caudo-rostral pattern was defined by the strongest LRP at the medulla and brainstem area, from where the pathology spreads through limbic areas to cerebral neocortex as the disease progresses.
(B) Amygdala-based pattern was defined by the strongest pathology in the amygdala or in limbic area and propagation to both caudal (brainstem, medulla, and spinal cord) and rostral neocortical regions.
(C) One subject could not be categorised as every brain region measured was scored as very severe.
Demographics [3] of the study subjects have been published previously as well as clinical [2], genetic [1] and neuropathological variables (SN neuronal loss [2], Braak NFT stage and Thal phases [5], CERAD score [3]).
Raw *p*-values are shown, asterisks show significance after Bonferroni correction *p<0.05, **p<0.01, ***p<0.001

Supplementary table 2. Results of the statistical analyses of Table 2.

Variable	Test	No LRP vs LRP in any brain region, no spinal LRP	No LRP vs LRP in any brain region and spinal LRP present	LRP in any brain region, no spinal LRP vs LRP in any brain region and spinal LRP present
Sex	Fisher's Exact Test	*p*=0.268795	*p*=0.123442	*p*=0.026066
Dementia	Fisher's Exact Test	*p*=0.055240	*p*=0.018749	*p*=1.000000
APOE4	Fisher's Exact Test	*p*=0.009912	*p*=0.352984	*p*=0.142523
SN neuron loss no, mild, moderate-severe	Fisher-Freeman-Halton Exact Test	*p*=0.043565	****p*=3.0015E-7	*p*=0.061376
Braak NFT groups 0-II, III-IV, V-VI	Fisher-Freeman-Halton Exact Test	**p*=0.000858	*p*=0.119758	*p*=0.106608
Thal phases 0, 1-3, 4-5	Fisher-Freeman-Halton Exact Test	*p*=0.106120	*p*=0.108159	*p*=0.858750
CERAD score groups none, sparse, moderate-frequent	Fisher-Freeman-Halton Exact Test	*p*=0.251884	*p*=0.243135	*p*=0.675982
Age at death	Independent-Samples Mann-Whitney U Test	*p*=0.529140	*p*=0.702269	*p*=0.435387
Age at dementia onset	Independent-Samples Mann-Whitney U Test	*p*=0.413925	*p*=0.794794	*p*=0.422494
Duration of dementia	Independent-Samples Mann-Whitney U Test	*p*=0.085759	*p*=0.868270	*p*=0.123344

Raw *p*-values are shown, asterisk(s) showing significant *p*-values <0.05 after Bonferroni correction (after Bonferroni correction **p*-value=0.02574, after Bonferroni correction ****p*-value= *p*=9.0045E-6).

Supplementary table 3. The characteristics of semiquantitative scores of spinal cord LRP in different anatomical regions

Spinal cord region	Total number of cases with spinal cord samples available n=303	Semiquantitative count mean (SD) of spinal cord LRP in all cases with brain LRP n=139	Semiquantitative count mean (SD) of spinal cord LRP in cases with concomitant brain and spinal cord LRP n=85
Cervical 6-7 dorsal	293	0.50 (0.901)	0.80 (1.036)
Cervical 6-7 ventral	295	0.81 (1.096)	1.30 (1.138)
Thoracic 3-4 dorsal	300	0.67 (1.044)	1.11 (1.148)
Thoracic 3-4 IML	301	1.20 (1.301)	1.96 (1.124)
Thoracic 3-4 ventral	301	0.72 (0.928)	1.18 (0.933)
Lumbar 3-4 dorsal	299	0.74 (1.097)	1.20 (1.187)
Lumbar 3-4 ventral	299	0.64 (0.924)	1.05 (0.987)
Sacral 1-2 dorsal	294	1.00 (1.232)	1.68 (1.188)
Sacral 1-2 ventral	296	0.76 (1.034)	1.27 (1.066)

Supplementary table 4. Results of the statistical analyses of Figure 1. Semiquantitative LRP score % of all cases with brain LRP. An ordinal regression random effect model with R was applied. LRP score % in dorsal C6-7 and in ventral C6-7 were used as references.

Dorsal	TH3-4 *p*-value (Estimate, Std. Error)	L3-4 *p*-value (Estimate, Std. Error)	S1-2 *p*-value (Estimate, Std. Error)
C6-7	***p*=0.000343[b] (1.511, 0.422)	****p*=1.06E-5[c] (1.855, 0.421)	****p*=1.43E-15[d] (3.904, 0.489)
Ventral	TH3-4	L3-4	S1-2
C6-7	*p*=0.19615 (-0.44960, 0.34783)	**p*=0.00239[a] (-1.11821, 0.36821)	*p*=0.92686 (-0.03223, 0.35111)

Raw *p*-values are shown, asterisk(s) show significant *p*-values < 0.05 after Bonferroni correction [a]*p*-value=0.01434, [b]*p*-value=0.002058, [c]*p*-value<6.36E-5, [d]*p*-value=8.58E-15).

Supplementary table 5. **The results of the statistical analyses of Figure 2.** Linear-by-Linear Association Mantel-Haenszel Test was used to assess the trend for increasing spinal cord LRP related to the DLB Consortium -types (olfactory-only n=15, amygdala-predominant n=10, non-class n=11, brainstem-predominant n=19, limbic n=41, diffuse neocortical n=43).

valid n	olfactory-only<amygdala-predominant<non-class<brainstem-predominant<limbic<diffuse neocortical
Cervical 6-7 ventral horn n=135	***p<0.001 p=2.6786E-11
Cervical 6-7 dorsal horn n=133	***p<0.001 p=2.3662E-7
Thoracic 3-4 ventral horn n=138	***p<0.001 p=2.3063E-9
Thoracic 3-4 intermediolateral column n=138	***p<0.001 p=4.8709E-11
Thoracic 3-4 dorsal horn n=137	***p<0.001 p=3.0773E-8
Lumbar 3-4 ventral horn n=136	***p<0.001 p=1.1138E-9
Lumbar 3-4 dorsal horn n=136	***p<0.001 p=5.6865E-10
Sacral 1-2 ventral horn n=136	***p<0.001 p=7.6389E-11
Sacral 1-2 dorsal horn n=134	***p<0.001 p=3.1436E-11

Raw *p*-values are shown. All *p*-values are significant after Bonferroni correction (all corrected *p*-values<0.001) and are marked with three asterisks.

Supplementary table 6. The results of the statistical analyses of Figure 4. The frequency of semiquantitative LRP score in spinal cord regions at four levels in dorsal horn and ventral horn, and at thoracic level IML of Caudo-rostral (n=83) vs. Amygdala-based (n=40) LRP type cases (Fisher's exact test).

Anatomic region	Caudo-rostral n=83 vs amygdala-based n=40 *p*-value (corrected *p*-value)
C6-7 ventral n=119	***p*=0.000573 (*p*=0.005157)
C6-7 dorsal n=117	*p*=0.007523 (*p*=0.067707)
TH3-4 ventral n=122	***p*=0.000142 (*p*=0.001278)
TH3-4 iml n=122	****p*=0.000003 (*p*=0.000027)
TH3-4 dorsal n=121	**p=0.000219 (*p*=0.001971)
L3-4 ventral n=120	**p*=0.003108 (*p*=0.027972)
L3-4 dorsal n=120	**p*=0.004638 (*p*=0.041742)
S1-2 ventral n=120	*p*=0.009432 (*p*=0.084888)
S1-2 dorsal n=118	****p*=0.000024 (*p*=0.000216)

Raw *p*-values are shown, asterisk(s) show significant *p*-values <0.05 after Bonferroni correction.

Supplementary table 7. Frequency of adrenal gland and lumbar dorsal root ganglion LRP compared with DLB Consortium LRP types and LRP progression patterns.

**LRP**		**Adrenal gland sample available (n=164)**		**Adrenal gland sample unavailable (n=140)**	**Lumbar dorsal root ganglion sample available (n=219)**		**Lumbar dorsal root ganglion sample unavailable (n=85)**
		**No**	**Yes**		**No**	**Yes**	
n		**142**	**22**		**200**	**19**	
	No LRP n=165[a]	93	0	72	121	0	44
**DLB Consortium LRP type**	Non-class n=11	6	0	5	6	0	5
	Brainstem-predominant n=19	11	1	7	12	0	7
	Olfactory-only[a] n=15	10	0	5	11	0	4
	Amygdala-predominant n=10	3	0	7	8	0	2
	Limbic n=41	9	4	28	28	4	9
	Diffuse neocortical n=43	10	17	16	14	15	14
**LRP progression pattern**	Caudo-rostral n=83	23	21	39	41	16	26
	Amygdala-based n=40	16	0	24	27	2	11
	All-highest n=1	0	1	0	0	1	0

Supplementary table 8. The results of statistical analyses of Figure 6.

a) Linear-by-Linear Association Mantel-Haenszel Test was used to assess the trend for increasing αSyn pathology in DRG and adrenal gland related to the DLB Consortium -types (olfactory-only n=15, amygdala-predominant n=10, non-class n=11, brainstem-predominant n=19, limbic n=41, diffuse neocortical n=43).

valid n	olfactory-only<amygdala-predominant<non-class<brainstem-predominant<limbic<diffuse neocortical
dorsal root ganglion n=98	*** p=0.000033
adrenal gland n=71	*** p=0.000006

Raw p-values are shown, asterisks show significant p-values < 0.05 after Bonferroni correction. All p-values are significant $p<0.001$ after Bonferroni correction.

b) Frequencies of αSyn pathology in DRG and adrenal gland in Caudo-rostral vs Amygdala-based LRP types (Fisher's Exact Test)

valid n	caudo-rostral n=83 vs amygdala-based n=40
dorsal root ganglion n=86	p=0.025702
adrenal gland n=60	***p=0.000427

Raw p-values are shown, ***p-value<0.0000854 after Bonferroni correction.

References

1 Myllykangas L, Polvikoski T, Sulkava R, Verkkoniemi A, Crook R, Tienari PJ, Pusa AK, Niinisto L, O'Brien P, Kontula K et al (1999) Genetic association of alpha2-macroglobulin with Alzheimer's disease in a Finnish elderly population. Ann Neurol 46: 382-390. https://www.ncbi.nlm.nih.gov/pubmed/10482269

2 Oinas M, Polvikoski T, Sulkava R, Myllykangas L, Juva K, Notkola IL, Rastas S, Niinisto L, Kalimo H, Paetau A (2009) Neuropathologic findings of dementia with lewy bodies (DLB) in a population-based Vantaa 85+ study. J Alzheimers Dis 18: 677-689. https://doi.org/10.3233/JAD-2009-1169

3 Polvikoski T, Sulkava R, Myllykangas L, Notkola IL, Niinisto L, Verkkoniemi A, Kainulainen K, Kontula K, Perez-Tur J, Hardy J et al (2001) Prevalence of Alzheimer's disease in very elderly people: a prospective neuropathological study. Neurology 56: 1690-1696. https://n.neurology.org/content/56/12/1690.long

4 Raunio A, Kaivola K, Tuimala J, Kero M, Oinas M, Polvikoski T, Paetau A, Tienari PJ, Myllykangas L (2019) Lewy-related pathology exhibits two anatomically and genetically distinct progression patterns: a population-based study of Finns aged 85. Acta Neuropathol 138: 771-782. https://doi.org/10.1007/s00401-019-02071-3

5 Savola S, Kaivola K, Raunio A, Kero M, Makela M, Parn K, Palta P, Tanskanen M, Tuimala J, Polvikoski T et al (2022) Primary age-related tauopathy in a Finnish population-based study of the oldest old (Vantaa 85+). Neuropathol Appl Neurobiol 48: e12788. https://doi.org/10.1111/nan.12788
